# Quercetin and Omega 3 ameliorate oxidative stress-induced neurodegeneration by Aluminium Chloride

**DOI:** 10.1186/1471-2164-15-S2-P45

**Published:** 2014-04-02

**Authors:** Haytham Abdallah, Mohamed Afifi, Aaser Mohamed Abdelazim

**Affiliations:** 1 Department of Biological Sciences, Faculty of Science, King Abdulaziz University, North Campus, PO Box 11508, Jeddah, 21463, Saudi Arabia; 2 Department of Biochemistry, Faculty of Veterinary Medicine, Zagazig University, Zagazig, Egypt

## Background

Background Exposure to high levels of Aluminium (Al) leads to a neurodegenerative disorders , which may be mediated through over generation of free radicals. So in the present study we investigated the ability of both Quercetin and Omega 3 to ameliorate Al adverse effect on brain antioxidant through monitoring the main brain antioxidant enzymes on molecular and cellular levels.

## Materials and methods

Forty male albino rats were used, they were divided into 4 groups; Control, Aluminum Chloride (AlCl3) supplemented group that orally supplemented with 100mg of AlCl3 (Sigma, St. Louis, MO) per Kg b.w. for two months. Quercetin group treated as ALCl3 group and orally supplemented with 100 mg/kg b.w. Quercetin for two months according to Hui et al. [1] and Omega 3 group that treated as ALCl3 and orally supplemented with 20 mg/kg b.w. Omega 3 for two months. At the end of the experiments, brain samples were taken and used for biochemical and molecular analysis.

## Results

**Figure 1:**
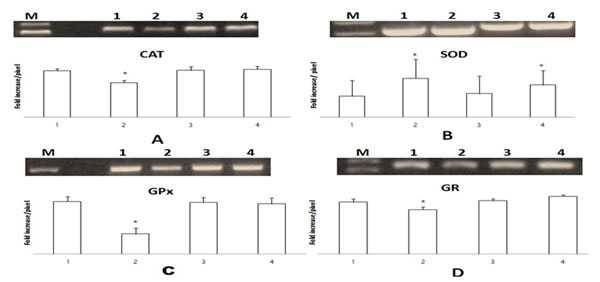
Expression level of mRNAs of (A) CAT, (B) SOD, (C) GPx and (D) GR genes in the brain tissue of rats. M: marker, 1: control group, 2: AlCl3 supplemented group, 3: Quecetin supplemented group and 4: Omega 3 supplemented group.

Our results indicate a significant increase in superoxide dismutase (SOD) activity and MDA level and a significant decrease in the activities of catalase (CAT), glutathione reductase (GR) and glutathione peroxidase (GPX) and levels of reduced glutathione (GSH) in brain tissues in AlCl3 supplemented group when compared with control or Quercetin and Omega 3 supplemented groups (Table 1). At the molecular level SOD mRNA showed the highest expression level in the AlCl3 supplemented group while the highest expression levels of mRNAs of CAT, GPx and GR was observed in the Quecetin and Omega 3 supplemented groups (Figure 1).

**Table 1:** Effects of ALCl3, Qurcetin and Omega 3 on antioxidants and MDA in brain tissues of rat.

Groups	GSH (umol/g tissue)	SOD (ug/g tissue)	CAT(µM H_2_O_2_ decomposed/g tissue)	GPx (µM /min/g tissue)	GR (unit/g tissue)	MDA (nmol /g tissue)
**Control**	86 ± 3^a^	0.45 ± 0.02^d^	1.66 ± 0.01^a^	51.4 ± 1.3^a^	19.3 ± 0.7^a^	5.4 ± 1^d^
**AlCl3 group**	53.7 ± 2.2^d^	0.93 ± 0.009^a^	1.5 ± 0.014^d^	26.7 ± 2.3^d^	9.9 ± 0.3^d^	29.9 ± 2.5^a^
**Quercetin group**	75.4 ± 2.9^b^	0.51 ± 0.007^c^	1.6 ± 0.02^b^	45.3 ± 0.6^b^	16.3 ± 0.7^b^	9.5 ± 1^c^
**Omega 3 group**	68.6 ± 3.2^c^	0.70 ± 0.016^b^	1.58 ± 0.01^c^	41.5 ± 0.9^c^	13.9 ± 0.4^c^	14.4 ± 2.5^b^

Means in the column carry different subscripts are significant at P<0.05

## Conclusions

Both Quercetin and Omega 3 has the ability to overcome the Al induced oxidative stress in brain, manifested by the significant reduction in free radicals concentration and induction of the activity and gene expression of the brain antioxidant enzymes.

## References

[B1] Hui L Lei Z Shaoping L Evaluation of antioxidant and immunity activities of quercetin in isoproterenol-treated rats Molecules 2012 15 4281 4291 10.3390/molecules17044281 PMC6268199 22491677

